# The Tapping-PROMS: A test for the assessment of sensorimotor rhythmic abilities

**DOI:** 10.3389/fpsyg.2022.862468

**Published:** 2023-01-16

**Authors:** Markus Georgi, Bruno Gingras, Marcel Zentner

**Affiliations:** ^1^Institute of Psychology, Teaching and Research Area of Work and Engineering Psychology, RWTH Aachen University, Aachen, Germany; ^2^Institute of Psychology, University of Innsbruck, Innsbruck, Austria

**Keywords:** sensorimotor synchronization, rhythm tapping, relative rhythm synchrony, rhythm perception, profile of music perception skills

## Abstract

Sensorimotor synchronization is a longstanding paradigm in the analysis of isochronous beat tapping. Assessing the finger tapping of complex rhythmic patterns is far less explored and considerably more complex to analyze. Hence, whereas several instruments to assess tempo or beat tapping ability exist, there is at present a shortage of paradigms and tools for the assessment of the ability to tap to complex rhythmic patterns. To redress this limitation, we developed a standardized rhythm tapping test comprising test items of different complexity. The items were taken from the rhythm and tempo subtests of the Profile of Music Perception Skills (PROMS), and administered as tapping items to 40 participants (20 women). Overall, results showed satisfactory psychometric properties for internal consistency and test–retest reliability. Convergent, discriminant, and criterion validity correlations fell in line with expectations. Specifically, performance in rhythm tapping was correlated more strongly with performance in rhythm perception than in tempo perception, whereas performance in tempo tapping was more strongly correlated with performance in tempo than rhythm perception. Both tapping tasks were only marginally correlated with non-temporal perception tasks. In combination, the tapping tasks explained variance in external indicators of musical proficiency above and beyond the perceptual PROMS tasks. This tool allows for the assessment of complex rhythmic tapping skills in about 15 min, thus providing a useful addition to existing music aptitude batteries.

## Introduction

Musical ability is a basic human endowment and efforts to discern its basic components go back at least a century (e.g., Shuter-Dyson and Gabriel, 1981; Sloboda, 1985). While one line of research has been concerned with basic processes underlying musical skills that are common to most individuals, another line of inquiry has devoted its attention to the assessment of individual differences in musical ability. Research along these lines has resulted in a number of test batteries for the detection of musical skills. Initially, these batteries were often developed in the context of music education, for example, to identify children who were sufficiently gifted to receive a formal music education. Tests such as Gordon (1995) Musical Aptitude Profile (MAP) or Wing (1948, 1968) Tests of Musical Ability and Appreciation are examples of this period in the development of musical aptitude tests (Zentner and Gingras, 2019).

More recently, musical ability tests have been developed to facilitate research on the determinants and development of musical ability, including its association with non-musical abilities such as reading, phonological awareness, second language abilities, memory, executive functions, as well as cognitive, emotional, and neurological deficits, such as dyslexia, dementia, or autism spectrum disorder. Examples include the Musical Ear Test (MET; Wallentin et al., 2010) or the Swedish Musical Discrimination Test (SMDT; Ullén et al., 2014; see Zentner and Gingras, 2019, for a review of these tests).

## Challenges in the development of music aptitude batteries

While the development of tools for assessing musical ability is promising, the range of musical skills captured by current psychometric tests tends to be limited to a circumscribed number of perceptual skills, usually acuity in the perception of rhythmic and tonal sequences, and, occasionally, of pitch discrimination (e.g., Wallentin et al., 2010; Ullén et al., 2014). However, music encompasses a much wider range of features, such as timbre, tuning, tempo, harmony, meter, etc. Skills in the perception of these latter features are not typically covered in batteries of general music aptitude (see Zentner and Gingras, 2019 for a review). Also, the extent to which performance on these tests generalizes to real-life expressions of musical ability is not always entirely clear (Zentner and Gingras, 2019). For example, most music exams or competitions are heavily geared toward an assessment of production skills, typically exhibited in recitals or performances.

### Trained vs. non-trained aspects of musical ability

A particular challenge in the development of tasks for the assessment of production skills is that these should assess the potential for acquiring production skills rather than assessing learned or trained production skills. Once individuals have received training in playing a musical instrument, the two aspects become inevitably intertwined. Still, tasks should ideally be devised in such a way as to be able to predict production abilities without requiring prior experience in musical production skills such as playing an instrument. Thus, musical production tasks ought to be at least conceived to be applicable to musically untrained individuals. Indeed, if some untrained individuals perform well on these tasks, perhaps as well as musically trained ones, this would indicate the presence of a high potential for acquiring production skills (i.e., a high level of musical aptitude), because training cannot explain their good performance. We have referred to these individuals as refer to these individuals as musical sleepers because of their existing, but dormant musical skills (Law and Zentner, 2012).

### Assessing music perception vs. music reproduction skills

Although musical production abilities usually require musical training, at a basic level, tapping can be performed also by untrained individuals. Tools for the assessment of both timing perception and tapping skills are at present limited in number and coverage. Iversen and Patel (2008) developed the Beat Alignment Test (BAT), in which participants are asked to detect whether beeps superimposed onto musical excerpts are on the beat or not in a perception task and to synchronize with the beat of the same excerpts in a production task. In the Harvard Beat Assessment Task (H-BAT; Fujii and Schlaug, 2013), paced tapping to music is complemented by tests focusing on the detecting/tapping the sequence of simple meters (duple vs. triple), sequences of tones with a tempo change, and the beat of patterns of time intervals.

The Battery for the Assessment of Auditory Sensorimotor and Timing Abilities (BAASTA; Dalla Bella et al., 2017) was conceived as a more comprehensive tool for the assessment of perceptual and sensorimotor timing skills. It includes various tasks aimed at differentiating individuals’ perceptual and reproductive timing skills, such as production tasks involving unpaced and paced tapping (with tones and music), synchronization-continuation, and adaptive tapping to a sequence with a tempo change. An interesting feature of some of these tests is the combination of perceptual and sensorimotor tasks using similar auditory stimuli, allowing examining the degree of overlap in perceptual and reproductive skills, but also eventual dissociations between perception and action in the timing domain.

According to the cognitive model of Peretz and Coltheart (2003), music is processed following a modular architecture in which the “input modules” (corresponding to auditory perception and analysis) are directly linked with the “output modules” associated with musical production skills such as singing and tapping. Importantly for our purposes, this model provides a framework in which perceptual abilities form the basis for production skills. Understanding the relationship between perceptual and production timing skills is important for several reasons.

First, it can shed light on the question whether perceptual and sensorimotor timing skills are based on the same underlying processes. Second, overlap may be of interest as an individual difference component, as a marker for specific timing impairments when perceptual and sensorimotor timing skills are dissociated, or as a potential indicator of special giftedness when the two are tightly associated. Third, the extent of overlap between perceptual and sensorimotor timing skills may assist researchers in deciding whether using one type of task may capture enough variance in timing-related skills in certain research contexts, such as when time with participants is very limited, when numerous other constructs must be assessed along with musical timing skills, or when these skills need to be included as a secondary or control variable.

Although research on the development of the above-mentioned batteries offers insights into these questions, the interpretability of the results is constrained by some limitations. For example, the perceptual and production timing tasks in the BAASTA yield different measurements (e.g., the perception tasks measure discrimination thresholds or sensitivity indices, while the production tasks measure tapping accuracy and variability). This makes it difficult to rule out differences in task properties as a factor determining associations between perceptual and reproduction tasks. Another limitation is that the reliability of the batteries is either unknown (Iversen and Patel, 2008; Fujii and Schlaug, 2013) or highly variable across perceptual subtests (Bégel et al., 2018), which can affect the size of observed correlations (e.g., Nunnally and Bernstein, 1994).

### Assessing rhythmic patterns vs. isochronous sequences

**Figure 1:**
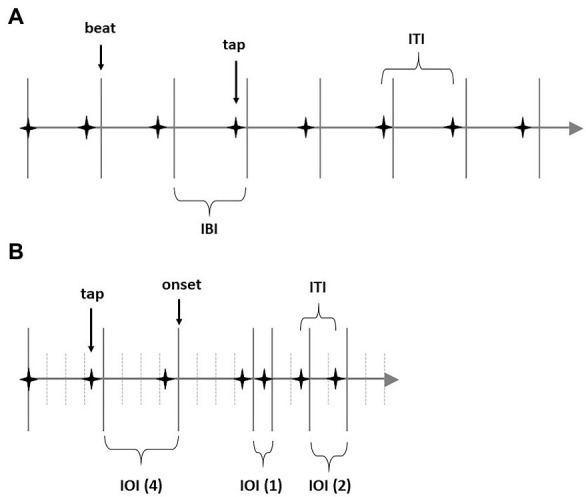
**(A)** Schematic representation of isochronous beat pattern (here with 8 beats). **(B)** Schematic representation of a rhythmic pattern (here with pattern 4:4:4:1:2:2). IBI, inter-beat-interval; ITI, inter-tap-interval; tap, finger press to synchronize a beat in **A**; IOI, inter-onset-interval; tap, finger press to synchronize with onset in **B**.

A more fundamental limitation of both BAASTA and the (H)-BAT is that the tasks used in these batteries focus mostly on the perception and reproduction of isochronous sequences, and that they are not specifically devised for testing the perception and reproduction of more complex rhythmic patterns. Although complex rhythms are typically gridded on an underlying isochronous pulse (at least in Western music), the perception of a regular beat involves the inference of an isochronous pulse given a repetitive stimulus, whereas rhythm perception requires an encoding of a pattern of durations (e.g., Fraisse, 1982; Clarke, 1999; Kotz et al., 2018). This distinction between isochronous beats and complex rhythmic patterns is illustrated in Figures 1A, B.

The accuracy of rhythm reproduction is considerably more complex to analyze than the reproduction accuracy of an isochronous beat sequence. Whereas the analysis of the latter can be based on circular statistics (Hasegawa et al., 2011), or rely on a calculation of mean absolute asynchronies between the taps and the pacing stimuli, these methods cannot be applied to determine the accuracy in rhythm reproduction due to the variable durations of sonic events in rhythmic patterns.

In the context of a study on the perception and production of syncopated rhythms, Fitch and Rosenfeld (2007) pioneered an approach in which participants heard a series of rhythmic patterns and were asked to repeat them by tapping. The participants’ tapping responses were digitized and compared to the correct tapping time on the basis of customized comparison and alignment software. The ability to reproduce rhythms was operationalized as the match between the correct time and the participants’ actual tapping time. More specifically, the authors computed matches between the anticipated and the reproduced rhythmic patterns *via* a cross-correlation of the two sets of rhythm intervals (ITIs compared to IOIs). There were some limitations with this approach as sometimes smaller cross-correlation actually led to more optimal matching (in 11 of 30 cases). It is worth noting, however, that Fitch and Rosenfeld’s goal was to address substantive questions about syncopated rhythm perception and reproduction. Thus, their methodology had to meet criteria that differ from those that are related to a systematic assessment of individual differences in rhythm reproduction abilities.

### Decomposing rhythmic reproduction accuracy: Synchrony and structural correctness

Among studies analyzing the ability to reproduce rhythmic patterns, we still see some important points missing, such as accurately assessing and separating two mathematical aspects of a reproduced rhythmic pattern, namely (a) synchrony by measuring reaction time deviation of the ITIs to the respective IOIs and (b) structural correctness of the tapping. Whereas asynchrony defines the average deviation of ITIs to the respective IOIs for a specific rhythm item, structural correctness corresponds to the full proportion of correctly tapped ITIs in relation to the IOIs indicated by the musical notation. For example, tapping two-quarter notes followed by one-eighth note would be structurally incorrect if the original rhythm started with the eighth note in the first position instead.

Concerning timing accuracy in rhythmic pattern reproduction, Moritz et al. (2013) measured the absolute deviance between ITI and IOI in milliseconds as the difference within each interval-per-interval alone, and not as the difference between each tap and its corresponding onset set in a whole timeline of a complete rhythm sequence. Thus, there is no attempt to measure rhythm as a whole time sequence, nor to assess the correctness of the rhythm structure.

### Limitations of previous work

There are studies focusing on structural correctness in performing rhythmic patterns but with no attention to timing accuracy. Keller and Burnham (2005) conducted a so-called rhythm canon task: The participant hears the consequent next to-be-reproduced rhythm during reproduction of the antecedent rhythm. Thus, the participant is distracted by a parallel task either memorizing the next rhythm, or reproducing a rhythm he had to memorize during continuous tapping before. In Keller and Burnham (2005), reproduction ability is only roughly measured by comparing binary codes in 150 ms steps: Tapping during a 150 ms sequence is operationalized to “1,” no tapping to “0.” The consequently binary code, e.g., “0-1-1-0-1” or “0-1-1-1-1,” will be compared with the normative code, e.g., “0-1-1-1-0.” Consequently, some binary codes (0-1-1-1-1) may be defined as more accurate than other binary codes (0-1-1-0-1).

Some studies used binary codes for assessing rhythmic pattern reproduction in a simpler manner than Keller and Burnham (2005), not requiring participants to memorize the next rhythm at the same time (rhythm canon). Concretely, Bonacina et al. (2021) as well as Tierney and Kraus (2015) had the same binary formula but worked with 200 ms instead of 150 ms sequences. Bonacina et al. (2020) did it with 250 ms sequences. Just 1 year before, the same authors Bonacina et al. (2019) did not mention the time interval used to measure the number of hit/false-alarm rates for respective drum or pause in their study. In any case, choosing an absolute time interval is arbitrary, whereas using binary codes to assess timing accuracy in rhythm reproduction provides only limited resolution.

Saito (2001) added *relative* values for the binary code formula, in addition to the absolute ones, but allowed a 15% deviation between response and reference interval to be counted as a correct tap. Even in respect to the self-chosen tempo for the rhythm item, this remains an arbitrary criterion. Further, Saito only counted the number of correct rhythms after performing this computation. A similar approach was used by Cameron and Grahn (2014) and Grahn and Schuit (2012), who both chose a 20% interval instead of a 15% one.

Here, we sought to distinguish more clearly between structural and deviation aspects in analyzing reproduced rhythms. Chen et al. (2008), and Matthew et al. (2016) did not choose an arbitrary deviation interval tolerance to be counted as a correct tap by taking 50% deviations which fills the whole rhythm sequence irrespective of the actual time interval of the IOI (quarter, eight, or sixteenth note), nor an absolute value such as the 150–250 ms deviations described above. But, as in Moritz et al. (2013), interval-per-interval was measured separately instead of measuring the rhythm sequence as a whole time sequence. Thus, incorrect rhythms with missed taps using this 50%-deviation criterion were included in the whole average formula. Further, Chen et al. (2008) arbitrarily decided to ignore the subsequent taps following the first tap within one onset-to-onset interval.

### The current study

To redress the above limitations in the assessment of rhythm reproduction, the current research aimed at developing a test for the assessment skills in the perception and sensorimotor reproduction of various target rhythms. To examine whether rhythm and isochronous tapping tasks draw on shared timing mechanisms, we also included tasks with isochronous taps. Accuracy of rhythm perception was determined by asking participants if two rhythmic patterns were same or different. In the production part, they were asked to reproduce the same rhythmic patterns. The ability to reproduce rhythmic patterns was analyzed both in terms of synchrony (inverted asynchrony as the average deviation of ITIs to the respective IOIs) and in terms of structural correctness of the rhythmic pattern. Sequences of isochronous pulses were presented at different tempi. In the perception part, participants were asked whether the tempo of the two sequences was same or different. In the production part, they were asked to reproduce isochronous tapping sequences at the same tempo.

### PROMS stimulus materials

In order to relate performance on production tasks to performance on perceptual tasks we used stimuli from the tempo and rhythm subtests of the Profile of Music Perception Skills (PROMS; Law and Zentner, 2012); thus, the name “Tapping-PROMS.” The PROMS exists in several versions that have all been shown to be both valid and reliable (Law and Zentner, 2012; Zentner and Strauss, 2017; Zentner and Gingras, 2019). Recent evidence includes demonstrations of large differences between musicians and non-musicians on Mini-PROMS test scores (e.g., Bosch et al., 2020; Sun et al., 2021), as well significant associations of PROMS-S scores with brain activation patterns involved in music processing (Rajan et al., 2021). Using perception and production stimuli from the same test source ensures that perceptual and production skills can be directly related to each other. Finally, we examined the extent to which variance in external indicators of musical ability, such as extent of musical training, may be explained by the included perceptual and production tasks.

The question of statistical power needs to be seen in the context of delivering a proof-of-concept with initial data for a new method to assess rhythmic reproduction skills rather than testing hypotheses relating to such skills. With that proviso in mind, we expected correlations between performance in perception and production tasks relating to comparable timing skills as well as validity correlations with external indicators of musical proficiency to be *r* ≳ 0.40 (Dalla Bella et al., 2017; Zentner and Strauss, 2017). G*power determined that to detect effects of this size with a power of 0.80 (*α* = 0.05), the sample size should be at least *N* = 40.

## Methods

### Participants

Forty participants (50% women), volunteered to participate in the study, ranging in age from 18 to 67 years (*M* = 28, *SD* = 10). Of these 40 participants, 20 participants (8 men, 12 women) volunteered to take part in a retest session around 1 week later (*M =* 7.35 days, *SD* = 0.67). Their age also ranged from 18 to 67 years (*M* = 30, *SD* = 12).

### Indicators of musical proficiency

In accordance with prior studies on the development of the PROMS (Law and Zentner, 2012; Zentner and Strauss, 2017), musical proficiency of the participants was assessed based on the self-reported number of years of musical training and the level of musicianship. Five levels of musicianship were provided as answer options (non-musician, music-loving non-musician, amateur musician, semi-professional musician, and professional musician), and participants selected the option that best described their level. Four of the 40 participants classified themselves as non-musicians, 20 as music-loving non-musicians, 15 as amateur musicians, and one as semi-professional musician. The two components, years of musical training and level of musicianship, were correlated highly with each other (*r* = 0.55, *p* < 0.001). Both were *z*-transformed and averaged to yield a musical proficiency score.

### Music perception battery (PROMS)

The perception of rhythm, tempo, pitch, and loudness was assessed using subtests of the PROMS (Law and Zentner, 2012), each consisting of 18 items. Rhythm and tempo were chosen as perceptual tasks matching the reproduction tasks (see below), whereas pitch and loudness were chosen to serve as unrelated control tasks, to which performance on the timing reproduction tasks should bear only a minimal relationship. All four subtests have shown good psychometric qualities (Law and Zentner, 2012), namely in internal consistency (*ω* = 0.72 to 0.80), test–retest reliability (*r* = 0.63 to 0.83), and convergent validity with other musical ability tests (*r* = 0.33 to 0.60).

A typical PROMS test item comprises a standard stimulus that is presented twice, followed by a comparison stimulus, which may or may not be identical to the standard stimulus, e.g., the comparison stimulus of a tempo item can be identical, or faster or slower than the (twice played) standard stimulus within a difference of 7 bpm (easy to distinguish) down to 1 bpm (difficult to distinguish). The participant has to identify whether the comparison stimulus is identical or not. The answer can be given at two different levels of confidence (“probably equal/different” or “definitely equal/different”). Participants receive one point for giving a confident correct answer (a correct hit or a correct rejection), and 0.5 point if the correct answer is given with lesser confidence. If the answer is skipped (“I do not know”) or wrong, 0 points are given (see Law and Zentner, 2012, for details). Before the beginning of each subtest, participants were given two practice items with feedback after each one.

### Rhythm reproduction (Tapping-PROMS)

The same PROMS items used to assess perceptual skills were also used in the rhythm reproduction task. As with the standard PROMS items, there was a time interval of 1.5 s between the standard stimulus and its repetition. The rhythm subtest items can be more or less difficult to reproduce, depending on the complexity of the standard stimulus. Following the PROMS, items were classified into easy (one or more notes added or subtracted on the downbeat), moderate (alterations on the upbeat notes), and complex (alterations at the sixteenth note-level; see Law and Zentner, 2012).

After listening to the standard rhythm or tempo stimulus for a second time (its repetition), participants were prompted to start reproducing it by a beep signal. The participants were instructed to reproduce the onsets of the standard stimulus by pressing the space bar with the index finger of their dominant hand. By pressing Enter, they proceeded to the next item. Before starting the actual task, three practice items were given in order to familiarize participants with the task.

### Tempo reproduction (Tapping-PROMS)

The tempo perception items of the PROMS were used for the tempo reproduction task, whereas the rhythm perception items were chosen for the rhythm reproduction task. In contrast to the rhythm items, the tempo items do not vary in their degree of complexity, but only in their isochronous pulse rate (beat). As in all PROMS subtests, the standard stimulus was played twice with a 1.5 s interstimulus interval. After a beep signal, the participants reproduced the beats of the tempo stimulus by pressing the space bar with the index finger of their dominant hand, as instructed before. With pressing Enter, they proceeded to the next tempo item. Before starting the actual task, three practice items were given in order to familiarize participants with the task before the actual testing items started.

### Procedure

**Figure 2:**
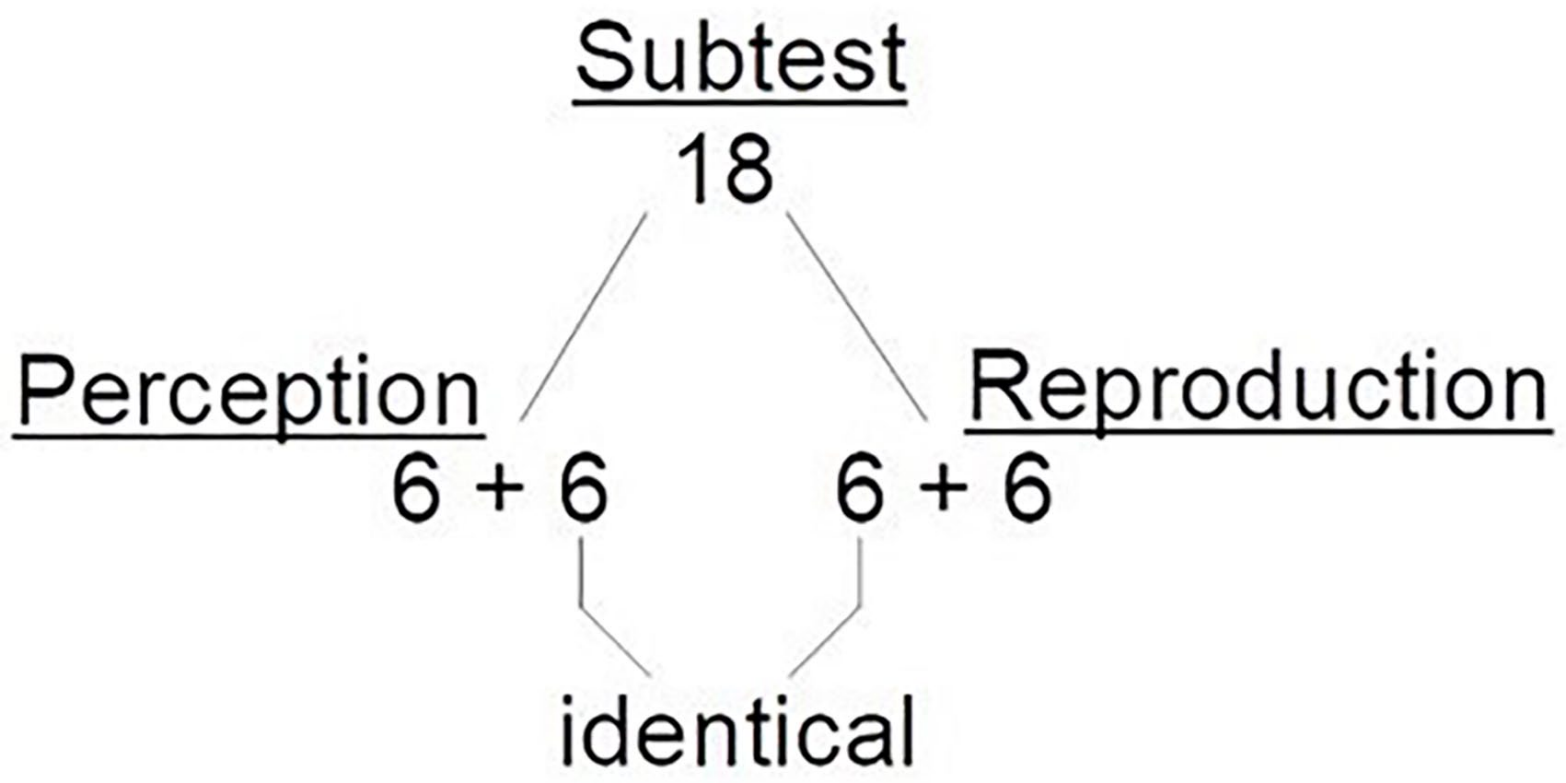
Distribution of the items per rhythm/tempo subtest of the PROMS. The current adaptation of the PROMS is depicted. The original battery comprises 18 items for each subtest, including 18 for the rhythm and 18 tempo subtests. In both subtests, 12 of the 18 stimuli were selected for the perceptual items, and 12 for the reproduction items. Of the 12 stimuli, 6 were identical between the perception and reproduction subtest of the same music dimension to control for possible training effects. Supplementary Table S1 provides a detailed overview over which 12 items were selected.

There were 12 items for the rhythm and 12 items for the tempo reproduction tasks. The stimuli were taken from the corresponding 18-item PROMS rhythm and tempo subtests, in such a way that 6 items were identical in the perception and reproduction version, and 6 items were different as illustrated in Figure 2. This trial allocation was chosen to make it possible to test for possible training effects, by comparing items that were the same and items that were different across the perceptual and reproduction tasks. Items of the rhythm and tempo reproduction subtests were presented in the same order as in the perceptual PROMS (see Supplementary material for more details). The 10-min Tapping-PROMS (rhythm and tempo reproductions tasks) was programmed and performed in Inquisit (Millisecond Software). Difficulty levels of the selected 12 items per subtest (rhythm, tempo, pitch, and loudness) were matched based on data from the PROMS (Law and Zentner, 2012).

Participants were tested individually in a laboratory at the Institute of Psychology of the University of Innsbruck. In an informed consent clause, participants were informed about the nature of the study and assured of the study’s anonymity and confidentiality and their right to withdraw from the study at any point. The experiment was administered on *Lenovo All-in-One PC ThinkCentre E93z—10CX002B (10CX002BGE)* computers, which include a set with Bluetooth mouse and keyboard. The participants opened a link in LimeSurvey (Version 2.05) on the computer screen to start the (online) experiment and put on headphones (Sennheiser HD 380 pro). The two subtests of the Tapping-PROMS (rhythm and tempo reproduction) and the perception subtests for rhythm, tempo, pitch, and loudness of the PROMS were administered according to the crossover design illustrated in Table 1. One session took about 45 min to complete.

**Table 1:** Sequences of administration of the tasks: reproduction tasks (bold); perception tasks—temporal (standard); and perception tasks—non-temporal (italicized).

	1	2	3	4	5	6
1:	**rhythm**	**tempo**	rhythm	tempo	|*pitch*	*loudness*
2:	*pitch*	*loudness*	|rhythm	tempo	**rhythm**	**tempo**
3:	**tempo**	**rhythm**	tempo	rhythm	|*pitch*	*loudness*
4:	*pitch*	*loudness*	|tempo	rhythm	**tempo**	**rhythm**
5:	**rhythm**	**tempo**	rhythm	tempo	|*loudness*	*pitch*
6:	*loudness*	*pitch*	|rhythm	tempo	**rhythm**	**tempo**
7:	**tempo**	**rhythm**	tempo	rhythm	*|loudness*	*pitch*
8:	*loudness*	*pitch*	|tempo	rhythm	**tempo**	**rhythm**

The numbers in the leftmost column correspond to the 8 sequences of administration, whereas the numbers in the topmost row refer to the position of a task within any given sequence. The pitch and tempo subtests, as well as the perception and reproduction subtests are always presented in one contiguous block.

### Computation of tempo reproduction accuracy

The calculation of the tempo reproduction accuracy was comparable to the way Dalla Bella et al. (2017) calculated the accuracy of isochronous taps. An outlier was defined as an ITI-value that was at least 3*IQR (inter-quartile-range) smaller than the first quartile or larger than the third quartile in the distribution of all ITIs of all participants in one item. When such ITI-outliers were present, and/or the participant produced more or less than the specified eight taps, the item was excluded from further analysis for this participant. After exclusion of these incorrect item reproductions, the series of ITIs (inter-tap-intervals) of the remaining item reproductions were directly linked to the series of IBIs (inter-beat-intervals). The tapping accuracy was determined in three steps. First, the absolute deviation in milliseconds of the taps from the corresponding beats was computed. Second, the ratio of these absolute deviations to the corresponding IBI was computed. Third, these ratios were converted into percentage values to obtain an average percentage value per item as the unit of measurement for tapping accuracy.

### Computation of rhythm reproduction ability

The accuracy of rhythm reproduction is considerably more complex to measure than the accuracy of tempo reproduction. First, it is not possible to apply tempo-matching algorithms to analyze the accuracy of rhythm reproduction because rhythmic patterns may be correctly reproduced at a tempo that differs from the target tempo (Honing, 2013). Indeed, a reproduced rhythmic pattern may substantially deviate from the reference in terms of absolute time, but still be structurally correct if the relative relations between the note values are preserved.

A second challenge lies in determining when a reproduced rhythmic pattern may be considered as being structurally correct or incorrect. By structurally incorrect we mean that note values have been fundamentally altered (say, from a quaver to a semi-quaver). Finally, the number of notes in the reproduced pattern may deviate from the number of notes in the reference pattern, for instance, because notes have been omitted or added. Since there are no ready-made timing matching algorithms that can identify structurally incorrect rhythm, in what follows, we will describe our approach for computing the ability of rhythmic pattern reproductions in some detail.

#### Identification of incorrect rhythm reproductions

To identify structurally incorrect rhythm reproductions, the series of ITIs per rhythm were checked with the given series of corresponding IOIs (inter-onset-intervals; see Table 2). If structural deviations were found in a rhythm reproduction (for instance, the temporal position of the ITI is closer to the temporal positions of subsequent IOIs than to these of the intended IOI in the rhythm; e.g., “518” in Table 2, middle row, is closer to the subsequent normative “520” than to the intended “260”) or the participant reproduced the incorrect number of taps anyhow, the item was treated as a structurally incorrect rhythm reproduction.

**Table 2:** Example for IOI series of a reference rhythm of the PROMS and three fictitious reproduction series, represented with millisecond values (upper part) and with a figure (lower part), to exemplify our approach to the assessment of rhythm tapping ability.

520	260	260	520	260	260	520	520	520		Normative IOI series
504	284	283	546	285	276	518	552	546		Correct rhythm
504	284	283	546	285	X	518	552	546	526	(structurally) Incorrect rhythm reproduction
504	284	283	708^*^	285	276	518	552	546		Incorrect rhythm reproduction (outlier)
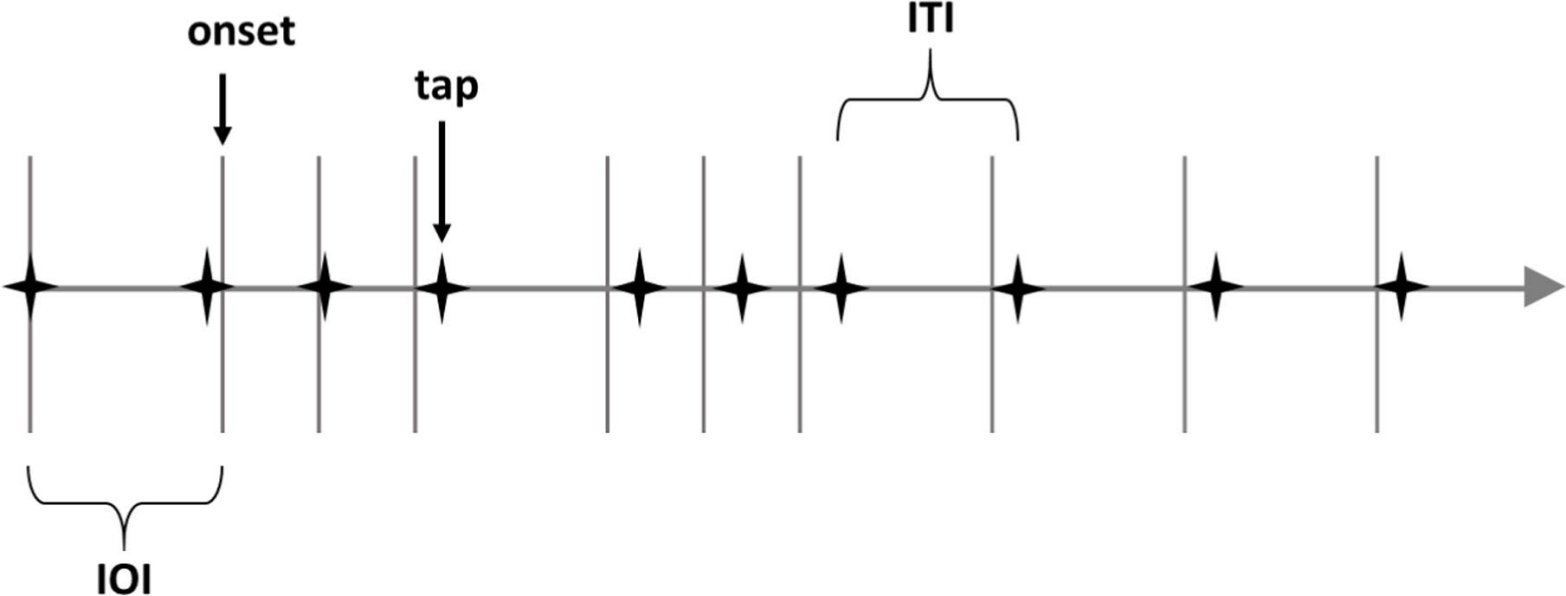											Correct rhythm
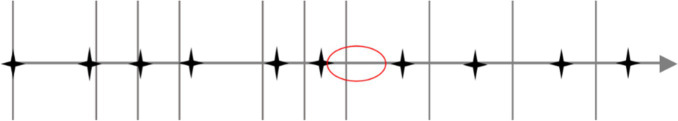											(structurally) Incorrect rhythm reproduction
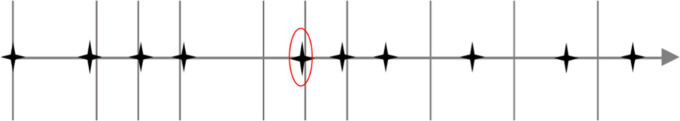											Incorrect rhythm reproduction (outlier)

Rhythm reproductions can be diagnosed as incorrect due to two types of errors, either due to a structurally incorrect pattern or due to the presence of one or more outliers. IOI, inter-onset-interval; ITI, inter-tap-interval; tap, finger press to synchronize with onset; X, gap after matching due to structural deviation. *Outlier. All values are indicated in milliseconds. Normative IOI numbers are in bold; incorrect or omitted ITIs are circled in red.

As with tempo, outliers among the ITIs were identified, following the same procedure as in Dalla Bella et al. (2017). Since the rhythmic patterns exhibited several different note durations, unlike the isochronous tempo patterns, this procedure was carried out separately on the distributions of the respective ITI-times within the structurally correct rhythm reproductions of one item. For example, in Table 2, the outlier analysis would be carried out for 520 ms (five columns) and for 260 ms (four columns) separately. Items containing one or more outliers or structural deviations, as well as items with an incorrect number of taps, were counted as incorrect rhythm reproductions and excluded from further analysis of their temporal synchrony.

Second, we considered how structurally accurate reproductions may be counted as correct regardless of the absolute tempo in which the pattern was reproduced. To this end, we computed the relative timing in addition to the absolute timing of the ITIs. Therefore, ITIs were transformed in a relative range from 0 for the first tap up to 1 for the last tap (e.g., see relative tap times in Table 3) and compared with relative IOIs (e.g., see numbers in bold in the last row of Table 3). Thus, structural deviations in relative rhythm reproduction were found the same way as in absolute rhythm reproduction but with relative values. Also, the outlier analysis in relative rhythm reproductions is similar to the outlier analysis in absolute rhythm reproductions and carried out separately on the distributions of the respective ITI-times but with relative values, e.g., for 0.1429 (four columns) and for 0.0714 (four columns) separately.

**Table 3:** Computation of accuracy of an absolute/relative rhythm (example of a correct rhythm reproduction taken from Table 2).

520	780	1,040	1,560	1820	2080	2,600	3,120	3,640	Absolute	Onset times
0.1429	0.2143	0.2857	0.4286	0.5	0.5714	0.7143	0.8572	1	Relative	
504	788	1,071	1,617	1902	2,178	2,696	3,248	3,794	Absolute	Tap times
0.1328	0.2077	0.2823	0.4262	0.5013	0.5741	0.7106	0.8561	1	Relative	
$\frac{16}{520}$	$\frac{8}{260}$	$\frac{31}{260}$	$\frac{57}{520}$	$\frac{82}{260}$	$\frac{98}{260}$	$\frac{96}{520}$	$\frac{128}{520}$	$\frac{154}{520}$	Absolute	Ratios of deviations to the normative IOIs
0.0101/**0.1429**	0.0066/**0.0714**	0.0034/**0.0714**	0.0024/**0.1429**	0.0013/**0.0714**	0.0027/**0.0714**	0.0037/**0.1429**	0.0010/**0.1429**	*	Relative	
3%	3%	12%	11%	32%	38%	18%	25%	30%	Absolute	Resulting deviations in percent
7%	9%	5%	2%	2%	4%	3%	1%	*	Relative	

The absolute (+/−) deviations in the dividends are obtained from the difference between the tap times and the respective onset times (see third row from the top). Subsequently, these absolute deviations are computed as a ratio of the respective inter-onset intervals (see lower rows). IOI, inter-onset-interval. Absolute deviations are measured in milliseconds, whereas relative deviations range between 0 and 1. Normative IOIs in bold.

* Not calculated, since a “1” in the relative rhythm had to be used as a measure for relative time scaling in the row.

Outliers or structurally incorrect rhythmic patterns are the two possible types of incorrect reproductions of both absolute and relative rhythms and were discarded from further calculation of temporal synchrony. Rhythms with no structural deviation or outlier, in turn, were counted as correct and investigated with respect to their temporal accuracy.

#### Calculation of the temporal accuracy of correct rhythms

In order to assess tapping accuracy in the case of correct rhythms, the absolute deviations (i.e., whether late or early) of the individual taps from their respective reference onsets were first measured in milliseconds. These absolute deviations were then computed as a ratio of the corresponding IOIs (see Table 3). Subsequently, these ratios were transformed into percentage values. The smaller the average deviation in percent, the better the tapping ability. Whereas absolute deviations were directly evaluated using raw timing data in milliseconds, for relative deviations the temporal position of the last tap was used as a reference point to evaluate relative time positions for all previous tap times in a time scale from 0 up to 1 before (see relative rhythms in Table 3).

As a final step, the ratios of deviations to the respective IOIs were used to compute the mean deviation per item in percent. As shown in the two lower rows of Table 3, the nine absolute deviation values yield a mean deviation of 19%, whereas the eight relative deviation values yield a mean deviation of 4%.

## Results

### Descriptive statistics

On average, 6 of 12 (50%; *SD* = 2.33) rhythm items were correctly reproduced (see Table 4 for further details). 10% of the rhythm reproductions were discarded due to outliers or wrong number of taps. The correct reproduction over all individual rhythm items ranged between 15% and 80%. No participant was able to reproduce all items correctly. The highest number of correctly reproduced rhythm items per participant was 10 (out of 12). The more complex the rhythms were, the poorer the synchronies of the taps tended to be. The mean synchrony (inverted asynchrony) value for tempo reproduction in one participant was more than 3 standard deviations from the mean and was thus identified as an outlier. Both this participant and another participant who reproduced all rhythms incorrectly were removed, reducing the sample size from *N* = 40 to *N* = 38.

**Table 4:** Means and standard deviations of the temporal deviations in the tempo reproduction and rhythm reproduction tasks of the Tapping-PROMS by task difficulty levels.

		Difficulty levels^*^		
		Easy	Moderate	Complex
Absolute rhythm synchrony	46 (17)	32 (16)	45 (28)	63 (44)
	Range 16 to 86	Range 7 to 59	Range 8 to 136	Range 13 to 219
Relative rhythm synchrony	23 (10)	12 (11)	20 (15)	36 (26)
	Range 6 to 45	Range 2 to 45	Range 2 to 72	Range 7 to 141
Percent correct rhythms	50%	76%	45%	38%
Tempo	25 (13)	–	–	–
	Range 6 to 69	–	–	–

Deviations (mean, standard deviation in parentheses, and ranging minimum and maximum values) of the taps to the respective beat/onset are given as percentages of the corresponding inter-beat-interval/inter-onset-interval. Written percentage values in the third row refer to the proportion of correct rhythm reproductions over whole sample. *N* = 38; except for moderate (*n* = 33) and complex (*n* = 32).

* The difficulty levels refer to the stimulus characterization as easy, moderate, and complex in the PROMS (Law and Zentner, 2012).

### Control of possible training effects

We examined possible training effects in all four reproduction subtests that could have occurred, for instance, if participants completed a temporal perception subtest before its reproduction equivalent (sequence effect) or due to the subset of overlapping items in the perception and reproductions tasks (item repetition effect, see Figure 2). Sequence effects were examined by comparing the experimental groups who completed the perception subtests *before* the reproduction tasks (N. 2, 4, 6, and 8 in Table 1) with the groups who performed the perception subtests *after* the reproduction tasks (N. 1, 3, 5, 7 in Table 1), using independent sample t-tests. Item repetition effects were investigated by comparing performance on repeated vs. non-repeated items using paired samples t-tests within the experimental group who performed the perception subtests first. No evidence was found for training effects, either for sequence effects [number of correctly reproduced rhythms: *t*(36) = 1.27, *p* = 0.21; absolute rhythm synchrony: *t*(36) = −1.89, *p* = 0.07; relative rhythm synchrony: *t*(36) = 0.39, *p* = 0.70; tempo synchrony: *t*(36) = −0.38, *p* = 0.71] or for item repetition effects [number of correctly reproduced rhythms: *t*(17) = 1.53, *p* = 0.14; absolute rhythm synchrony: *t*(17) = −0.75, *p* = 0.47; relative rhythm synchrony: *t*(17) = −0.70, *p* = 0.50; tempo synchrony: *t*(17) = −1.96, *p* = 0.07].

### Reliability

#### Internal consistency of rhythm and tempo reproduction subtests

We used McDonald’s omega rather than Cronbach’s alpha as an estimate of internal consistency, because of the more realistic assumptions underlying omega (Dunn et al., 2014). The internal consistency of the isochronous tempo items was *ω* = 0.94. The internal consistency of relative and absolute asynchronies of the rhythm items could not be directly determined given that a significant portion of the rhythmic patterns were not reproduced correctly. To circumvent this problem, we imputed values as the mean value per item over all participants, when they were missing. This way of handling missing data has been found provide acceptable approximations to full data sets (Shrive et al., 2006).

Absolute/relative rhythm synchrony subtests yielded values of *ω* = 0.44 and *ω* = 0.58, respectively. The internal consistency estimate for the number of correct rhythms (see Table 2) was based on a dichotomous correct/incorrect coding and yielded a value of *ω* = 0.58. In evaluating the size of the respective coefficients, it needs to be considered that internal consistency coefficients ≈ 0.60 are acceptable in research contexts (e.g., Aron and Aron, 1999; Hair et al., 2006).

#### Test–retest reliability of rhythm and tempo reproduction subtests

Because single observations can affect the correlation disproportionately in a sample of this size, we report intra-class and Spearman rather than Pearson’s correlations. The values were as follows: tempo synchrony, *ICC* = 0.90, *ρ*(18) = 0.78, *p* < 0.001; absolute rhythm synchrony, *ICC* = 0.77, *ρ*(18) = 0.60, *p* < 0.01; relative rhythm synchrony *ICC* = 0.73, *p* < 0.001, *ρ*(18) = 0.45, *p* < 0.05; and number of correctly reproduced rhythm items, *ICC* = 0.85, *ρ*(18) =0.74, *p* < 0.001.

### Convergent and discriminant validity

To evaluate validity, we examined whether the pattern of convergent and discriminant associations conformed with what could be reasonably expected. Specifically, we expected performance in rhythm tapping to be more strongly correlated with performance in rhythm perception than in tempo perception. Conversely, we expected performance in tempo tapping to be more strongly correlated with performance in tempo perception than in rhythm perception. Furthermore, we expected both tapping tasks to be only marginally correlated with non-temporal perception tasks, pitch, and loudness.

**Figure 3:**
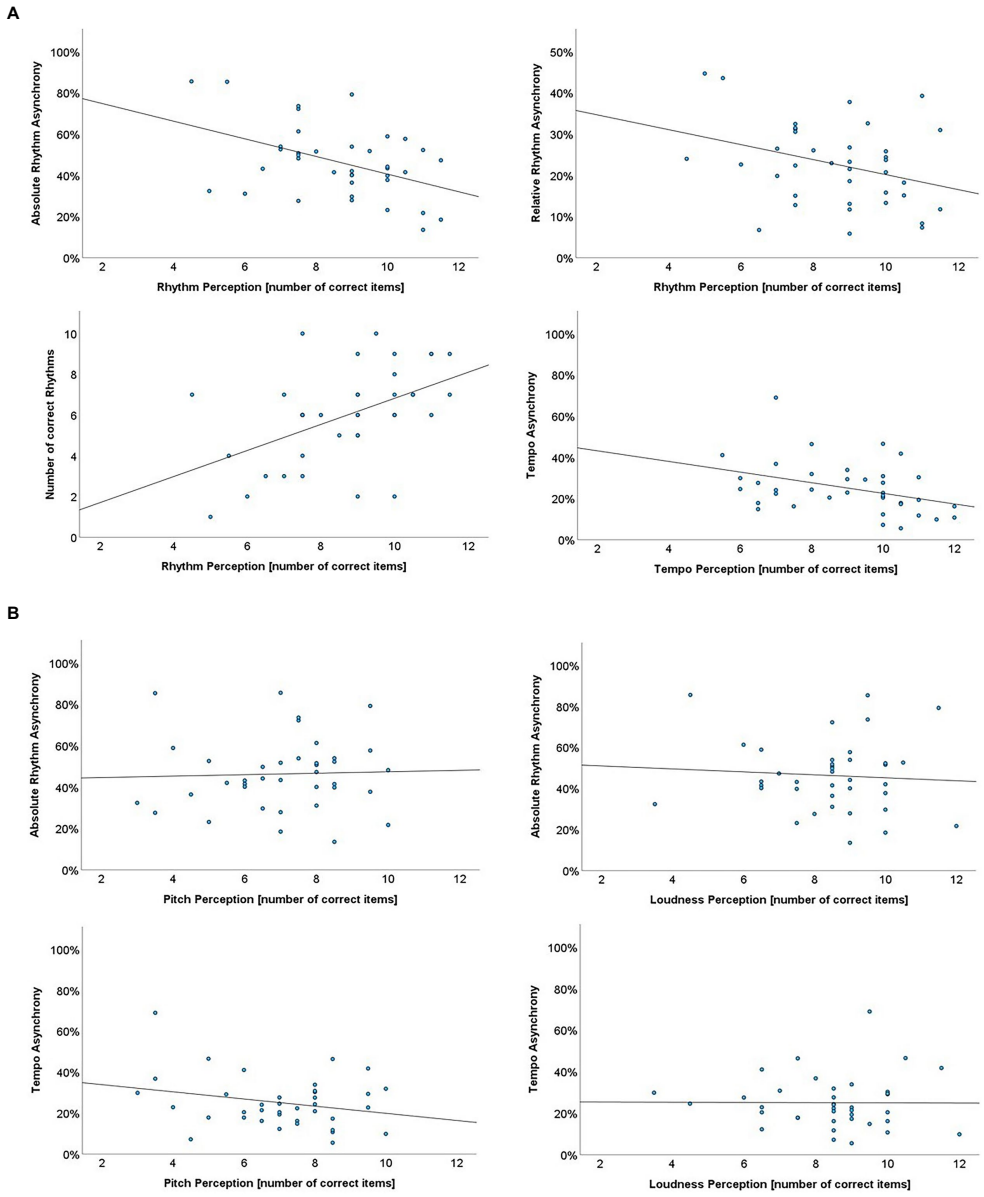
**(A)** Scatterplot visualization of the relationship between performance on rhythm and tempo reproduction tasks with performance on the respective corresponding perceptual tasks. **(B)** Scatterplot visualization of the relationship between performance on rhythm (absolute rhythm asynchrony) and tempo reproduction tasks with performance on the non-temporal perception tasks.

Overall, the pattern of convergent and discriminant correlations matched these predictions. As shown in Table 5, synchrony of tempo reproduction was sizably correlated with accuracy of tempo perception (see Figure 3), but not with accuracy of rhythm perception, as would be expected. The ability to tap to rhythmic patterns, in turn, was correlated significantly with accuracy of rhythm perception, regardless of the rhythm reproduction metric used, as shown also in Figure 3. Performance in the timing-irrelevant pitch and loudness perception subtests was not significantly correlated with performance on any timing reproduction tasks (see Figure 3B for absolute rhythm and tempo asynchrony). Although these differences cannot be overinterpreted due to the relatively small sample size, it is worth noting that the pattern of convergent and discriminant associations conforms with theoretical expectations.

**Table 5:** Correlation between PROMS perception subtests (left column) and rhythm and tempo reproduction tasks (upper row). The correlations between accuracy of tempo reproduction and the three measures of rhythm reproduction ability are reproduced in the bottom row.

		Reproduction			Tempo	Rhythm			Total correct	Absolute	Relative
Perception	Rhythm	**0.50** ^**^	0.44^**^	0.33^*^	0.3
	Tempo	0.35^*^	0.38^*^	0.17	**0.38** ^*^
	Pitch	*0.19*	*−0.04*	*0.24*	*0.26*
	Loudness	*0.31*	*0.07*	*0.12*	*0.01*
	Tempo Reproduction	0.03	0.40*	0.35*	*-*

Total correct, total number of correctly reproduced items; absolute, rhythm synchrony (inverted asynchrony) in absolute time; relative, rhythm synchrony (inverted asynchrony) in relative time. Convergent validity correlations boldfaced; discriminant validity correlations italicized. *N* = 38. ^*^p < 0.05; ^**^p < 0.01.

### Criterion and incremental validity

Although the current test should be able to assess variations in rhythm reproduction abilities in non-musicians, overall, musicians ought to perform better on the test than non-musicians due to their extensive training. Therefore, extent of training ought to correlate with test performance. Accordingly, examining variance explained in musicianship level as an external criterion of musical proficiency by test performance components seems a plausible way to assess criterion validity.

To this end, we ran a hierarchical regression using the composite index of musical proficiency (see Methods) as the outcome, and the various perceptual and reproduction tests as predictors. Next to tempo reproduction, we added absolute rhythm synchrony and number of correct rhythms as predictors but not *relative* rhythm synchrony due to the high correlation between absolute and relative synchrony (*r* = 0.59, *p* < 0.001). The amount of variance in the musical proficiency index explained by the four perception subtests was *R^2^adjusted* = 0.19, *F*(4, 33) = 3.18, *p* < 0.05, but significantly increased to *R^2^adjusted* = 0.35, *F*(7, 30) = 3.79, *p* < 0.01 (*F*change = 3.61, *p* < 0.05), when the new tapping reproductions tasks were added. Rhythm tapping (number of correctly reproduced rhythms, and absolute rhythm synchrony) alone significantly increased variance explained beyond the perceptual subtests to *R^2^adjusted* = 0.32, *F*(6, 31) = 3.85, *p* < 0.01 (*F*change = 4.04, *p* < 0.05), whereas tempo tapping alone did not (*F*change = 1.16, *p* = 0.29). This pattern of findings suggests that external criteria of musical proficiency were better predicted by a combination of tapping and perception tasks, and that improvement in prediction was largely attributable to the new rhythm tapping task.

## Discussion

The PROMS was originally developed as a test battery for assessing music perception skills. The current study extended the PROMS by adding the Tapping-PROMS, a standardized sensorimotor reproduction test. The Tapping-PROMS contains several rhythm and tempo items designed to be reproduced by finger tapping. With this new addition to the PROMS test battery, it is now possible to assess the ability to tap to rhythmic patterns of various difficulty levels in 15 min or less.

The new rhythm reproduction measurement in particular cuts a path into the largely uncharted territory of assessing abilities in reproduction of complex rhythmic patterns, as opposed to the well-established assessment of timing skills when processing isochronous pulse sequences. In devising this measure, we considered both the structural correctness of the IOI sequence and the time-related component in rhythm tapping. Thus, the measurement consisted of three rhythm reproduction components, i.e., number of correctly reproduced items, absolute rhythm synchrony, and relative rhythm synchrony.

An important consideration in constructing the new measure was to explore different metrics and determine their relative merits based on their reliability and validity. For example, the reliability of number of correctly reproduced items, absolute rhythm synchrony, and tempo tapping synchrony was within the bounds of acceptability, whereas the test–retest reliability for relative rhythm synchrony was not. Internal consistency was lower than test–retest reliability for number of correctly reproduced items. This could be due either to the greater consistency of performance in the retest-sample or to the limited amount of information contained in dichotomous data.

With regard to validity, the pattern of convergent and discriminant associations with performance on rhythm reproduction conformed to theoretical expectations overall, particularly if the number of correctly reproduced items and absolute synchrony are used as performance metrics. Thus, all measures of rhythm reproduction were correlated with rhythm perception, as would be expected since both are related to rhythm as one and the same music dimension. Further, two measures of rhythm reproduction (number of correctly reproduced items and absolute synchrony) were also correlated with tempo perception. This might be because tempo is a core component of rhythm (Honing, 2013). Tempo reproduction, in turn, was correlated only with tempo perception, which is consistent with the findings by Dalla Bella et al. (2017) who found several tempo perception and tempo reproduction tasks to be significantly correlated. Rhythm perception was not associated with better performance on the tempo reproduction subtest, presumably due to the absence of complex rhythmic elements in isochronous beats. Pitch and loudness perception subtests, as non-temporal perception subtests, were not significantly correlated with the temporal reproduction scales, indicating discriminant validity.

Against an overall convergent validity pattern, two findings do not have an obvious interpretation. First, the correlations with the relative synchrony metric, though conforming to expectations in most cases, were somewhat weaker than with the number of correct rhythms and absolute synchrony metric. In principle, the idea of deriving a relative time measure to dissociate rhythm from tempo perception and reproduction seems sound. However, it is possible that the transformation of absolute time values, measured in milliseconds, into a relative time measure between 0 and 1 discarded too much information.

Second, the absence of a significant association between performance on the tempo reproduction subtest and the number of correctly reproduced rhythm items was somewhat surprising, especially when considering that performance on the tempo reproduction task was significantly correlated with performance on the rhythm reproduction task when measured in absolute or relative synchrony. One possible explanation is that, whereas the number of correctly reproduced items was a count of correctly reproduced items against all reproduced items, including structurally incorrect ones, absolute or relative synchrony was only measured on items that were structurally correct. If this interpretation is correct, it indicates that tempo-timing skills alone cannot predict whether individuals are capable of correctly reproducing a complex rhythmic pattern, although it does predict the accuracy of timing in correctly reproduced rhythms.

With regard to criterion validity, prediction of external musical proficiency measures was significantly improved by adding the Tapping-PROMS to the perceptual PROMS subtests. However, the improvement was largely due to rhythm rather than tempo tapping. The finding that rhythm reproduction explains more variance in external musical proficiency than tempo reproduction is noteworthy in light of the relative paucity of studies on rhythm tapping as compared to isochronous beat tapping (Repp, 2005; Repp and Su, 2013). One possible explanation is that rhythmic patterns contain more complex musical information than isochronous beats, such as varying note durations at different complexity levels. The correct reproduction of rhythms may therefore require more advanced sensorimotor temporal skills and therefore be a more sensitive indicator of musical ability than beat tapping.

## Limitations

The contribution of this research needs to be interpreted in the light of its limitations. First, we do not suggest that the new rhythm reproduction task and the methods for analyzing it represent the only suitable approach determining rhythm reproduction skills. For example, our test could be viewed as Western-centric, since it makes use of complex rhythms typically found in Western music but not necessarily in other musical cultures. Along similar lines, the items used binary rhythms. Though reasonable as a starting point, future revisions of the battery should include a greater variety of rhythms.

Second, in the absence of objective standards for determining when a rhythmic figure is reproduced correctly, we used several approaches to data processing and analysis. Although the methods provided consistent findings overall, the computation of relative asynchronies led to results that were less compelling than might have been expected. Other methods, in particular common time-series methods such as dynamic time warping, or recent extensions such as Time Alignment Measurement (Müller, 2007; Folgado et al., 2018), allow for a continuous measure of rhythmic accuracy and are flexible enough to handle time series of different lengths (for instance, participants tapping at different tempi). However, these methods do not identify structural inaccuracies (for example, missing taps), which are a crucial aspect when analyzing rhythm reproduction accuracy.

Third, the current study’s sample size and the extent of validation were modest, but this limitation should be seen in the context of the aim of the current research, which was to introduce a novel method for assessing of rhythm reproduction skills, rather than the examination of specific hypotheses relating to such skills. Furthermore, it is not unusual to champion new methods requiring labor-intensive scoring procedures on samples of this size (see Dalla Bella et al., 2017, for a recent example). Still, caution is warranted in interpreting the results. Future research should seek to establish associations with a broader array of related tests, such as the BAASTA, or the *Goldsmiths Musical Sophistication Index* (Gold-MSI; Müllensiefen et al., 2014). It also bears mentioning that the sample of participants cannot be regarded as representative. As a result, the extent to which our findings generalize to other types of populations is a matter for future research.

Fourth, the manual scoring procedure for the rhythm reproduction tasks is rather a labor intensive, which can limit the usefulness of the test. To circumvent this limitation, we wrote an R script that is able to automatically analyze the data. The results obtained with the R script are in agreement with the results obtained using a manual approach. This script is available from the dedicated OSF website,^1^ next to Tapping-PROMS applications in both Inquisit and PsychoPy/Python (freeware on https://www.psychopy.org/download.html). In future, this Tapping-PROMS application will be accessible through the PROMS website.^2^

The Tapping-PROMS was originally programmed in Inquisit, as it is a recommended software to capture reaction time measurement data online (de Clercq et al., 2003; Schubert et al., 2013; Kulikowski and Potasz-Kulikowska, 2016). However, we also created a PsychoPy version of the Tapping-PROMS, as PsychoPy as a freeware has comparable precision in reaction time measurements (Bridges et al., 2020).

## Conclusion

After a period in which the focus of musical aptitude tests was on musical perception skills, there are now increasing efforts to also capture musical reproduction abilities (Zentner and Gingras, 2019). These efforts are in their early stages, and they are limited in several ways. Still, they represent a critical point of departure from which a more comprehensive assessment of musical ability that approximates the actual process of making music can be developed (Levitin, 2012). Beyond their contribution to the research literature, the rhythm production test introduced here could play a role in the diagnosis of neurodevelopmental and neurodegenerative disorders. For example, impairments in rhythm tapping have been found to play a more significant role in predicting children’s phonological awareness than beat tapping (Flaugnacco et al., 2015). The early identification of impairments in rhythm reproduction could thus be useful in devising training programs for the prevention of reading difficulties. Furthermore, neural and perceptual aspects of rhythm processing that are disrupted in ADHD patients (e.g., decreased motor connectivity within neuromotor areas, inconsistent sensorimotor timing linked to inhibitory control) are particularly well developed in musicians (Slater and Tate, 2018), suggesting a possible role for the current tasks in assessing areas for improvement in ADHD. Similarly, the current tempo and rhythm tasks as a whole might be helpful in identifying prodromal markers of Parkinson’s disease and help particularizing rehabilitation based on timing impairment profiles (Wearden et al., 2008; Spaulding et al., 2013).

While more research on the neuroscience of components of rhythm perception and production is needed to determine the value of such potential applications, our findings suggest that rhythm reproduction tasks provide information on musical ability that goes beyond the information that can currently be gained from existing music perception and tempo reproduction tests.

## Open practices statement

Tapping-PROMS applications in both Inquisit and PsychoPy/Python, an algorithm key file for manually detecting rhythmic structural deviations and measuring asynchronies, and an R script for automatically analyzing data output files are available on the OSF website (https://osf.io/df2gr/). The study was not preregistered.

## Data availability statement

The raw data supporting the conclusions of this article will be made available by the authors, without undue reservation.

## Ethics statement

Ethical review and approval was not required for the study on human participants in accordance with the local legislation and institutional requirements. The patients/participants provided their written informed consent to participate in this study.

## Author contributions

MG, BG, and MZ conceived the presented idea. MG gathered the data, performed the statistical analysis, wrote the first draft of the manuscript. BG and MZ wrote sections of the manuscript. All authors contributed to the article and approved the submitted version.

## Conflict of interest

The authors declare that the research was conducted in the absence of any commercial or financial relationships that could be construed as a potential conflict of interest.

## Publisher’s note

All claims expressed in this article are solely those of the authors and do not necessarily represent those of their affiliated organizations, or those of the publisher, the editors and the reviewers. Any product that may be evaluated in this article, or claim that may be made by its manufacturer, is not guaranteed or endorsed by the publisher.

## Supplementary material

The Supplementary material for this article can be found online at: https://www.frontiersin.org/articles/10.3389/fpsyg.2022.862468/full#supplementary-material

## Footnotes

^1^ https://osf.io/df2gr/

^2^ https://www.uibk.ac.at/psychologie/fachbereiche/pdd/personality_assessment/proms/use-the-proms-in-your-own-research/index.html.en
