# Supplementary material for: The Tapping-PROMS: A test for the assessment of sensorimotor rhythmic abilities
Source: Front Psychol. 2023 Jan 16;13:862468. doi: 10.3389/fpsyg.2022.862468 (PMC9886312; doi:10.3389/fpsyg.2022.862468)
Supplement: Supplementary file 1 [file Data_Sheet_1.PDF]

Supplementary Table

PROMS items used in the current study

	PROMS Subtests					
Item Number	**Rhythm Tapping**	**Rhythm Perception**	**Tempo Tapping**	**Tempo Perception**	**Pitch Perception**	**Loudness Perception**
1	-	**R1**	**T1**	**T1**	-	-
2	-	**R2**	**T2**	-	**P2**	**L2**
3	**R3**	**R3**	-	**T3**	**P3**	**L3**
4	**R4**	-	**T4**	-	-	**L4**
5	**R5**	**R5**	-	**T5**	**P5**	**L5**
6	**R6**	**R6**	-	**T6**	**P6**	**L6**
7	-	**R7**	**T7**	**T7**	-	-
8	**R8**	-	**T8**	-	**P8**	**L8**
9	**R9**	-	**T9**	-	**P9**	-
10	**R10**	-	**T10**	**T10**	**P10**	**L10**
11	**R11**	-	**T11**	**T11**	**P11**	**L11**
12	**R12**	**R12**	**T12**	**T12**	**P12**	**L12**
13	-	**R13**	**T13**	-	**P13**	-
14	**R14**	-	**T14**	**T14**	**P14**	**L14**
15	**R15**	**R15**	-	**T15**	-	**L15**
16	**R16**	**R16**	**T16**	-	-	-
17	-	**R17**	-	**T17**	-	**L17**
18	-	**R18**	-	**T18**	**P18**	-

Note. Shown above are the 12 items selected from the respective 18-item subtests of the original PROMS (bold font). Omitted items are represented with a dash (-). All items are part of the publicly accessible PROMS.* For convenience the audio files for all items of the rhythm and tempo subtests are also accessible via the OSF website and are labelled using the same letters and numbers as in the table above (https://osf.io/df2gr/).

R = Rhythm; T = Tempo; P = Pitch; L = Loudness

* https://www.uibk.ac.at/psychologie/fachbereiche/pdd/personality_assessment/proms/take-the-test/
